# Art and theatre for health in rural Cambodia

**DOI:** 10.1080/11287462.2017.1411762

**Published:** 2017-12-07

**Authors:** Chea Nguon, Lek Dysoley, Chan Davoeung, Yok Sovann, Nou Sanann, Ma Sareth, Pich Kunthea, San Vuth, Kem Sovann, Kayna Kol, Chhouen Heng, Rouen Sary, Thomas J Peto, Rupam Tripura, Renly Lim, Phaik Yeong Cheah

**Affiliations:** ^a^ National Center for Parasitology, Entomology and Malaria Control, Phnom Penh, Cambodia; ^b^ Provincial Health Department, Battambang, Cambodia; ^c^ Provincial Health Department, Pailin, Cambodia; ^d^ Mahidol Oxford Tropical Medicine Research Unit, Faculty of Tropical Medicine, Mahidol University, Bangkok, Thailand; ^e^ Nuffield Department of Clinical Medicine, University of Oxford, Oxford, UK; ^f^ Faculty of Medicine, University of Amsterdam, Amsterdam, The Netherlands; ^g^ Quality Use of Medicines and Pharmacy Research Centre, School of Pharmacy and Medical Sciences, University of South Australia, Adelaide, Australia; ^h^ The Ethox Centre, University of Oxford, UK

**Keywords:** Art, Cambodia, community engagement, malaria, theatre

## Abstract

This article describes our experience using art and theatre to engage rural communities in western Cambodia to understand malaria and support malaria control and elimination. The project was a pilot science–arts initiative to supplement existing engagement activities conducted by local authorities. In 2016, the project was conducted in 20 villages, involved 300 community members and was attended by more than 8000 people. Key health messages were to use insecticide-treated bed-nets and repellents, febrile people should attend village malaria workers, and to raise awareness about the risk of forest-acquired malaria. Building on the experience and lessons learnt in the year prior, the 2017 project which was conducted in 15 villages involved 600 community members and attracted more than 12,000 people. In addition to the malaria theme, upon discussion with local health authorities, secondary theme (infant vaccination) was added to the 2017 project. We learnt the following lessons from our experience in Cambodia: involving local people including children from the beginning of the project and throughout the process is important; messages should be kept simple; it is necessary to take into consideration practical issues such as location and timing of the activities; and that the project should offer something unique to communities.

## Introduction

Countries in Southeast Asia are working towards the elimination of *Plasmodium falciparum* malaria (WHO, 2015). In rural Cambodia, a village malaria worker (VMW) programme provides free diagnosis and treatment (Canavati et al., 2016). The success of the programme depends on the sustained motivation of isolated health workers and on local communities choosing to make use of the services. However, in remote communities there is a need to improve awareness and understanding about malaria and educate people about the government’s elimination activities (National Center for Parasitology, 2016). Art and theatre engagement approaches could be useful as many people are illiterate and do not attend village meetings to receive health education. Khmer (Cambodian) traditional drama uses comedy and music to tell stories that resonate with villagers’ lives and this art form is popular in rural communities (Lim, Peto, Tripura, & Cheah, 2016). This article describes our experience in 2016–2017 using art and theatre to engage rural communities to understand malaria and support malaria control and elimination in western Cambodia.

## Setting

Village populations ranged from approximately 150 to 3000 people with an average of 800 people in each village. The villages are generally poor and most families are involved in small-scale agriculture. The villages typically have only basic infrastructure: poor access to potable water, sanitation and irrigation systems, limited electricity supplies, minimal post-primary education, and poor access to health services. During sunny days, villagers commute on foot or using motorbikes. Several hours of heavy rainfall and commuters can be stranded because rivers rise so high as to destroy bridges. Locally, the presence of millions of hidden and unexploded land mines during the Khmer Rouge regime in the 1970s and in the civil war in the 1980s makes the local journey difficult and many villagers have lost legs from landmine blasts.

## Art and theatre for health project

The project was a pilot science–arts initiative to supplement existing engagement activities conducted by local authorities and a community engagement team supporting a malaria elimination study (clinicaltrials.gov NCT01872702). It was a collaboration between the Mahidol Oxford Tropical Medicine Research Unit (MORU), the Battambang and Pailin Provincial Health Departments and the Cambodian National Centre for Parasitology, Entomology and Malaria Control (CNM). In 2016, our engagement project had three key messages: (1) to use insecticide-treated nets and repellents (2) to consult a village malaria worker to get early diagnosis and treatment, and (3) to raise awareness about the risk of malaria in local forests. In 2017, upon discussion with local health authorities secondary theme, infant vaccination, was added.

The provincial health authorities selected a local drama group based on a competitive process. The selected group were trained on malaria and the key malaria-related messages (and infant vaccination in the second year). Local health staff supported the drama group to develop performance routines and scripts containing key messages about malaria (Guyant et al., 2015). Villages were selected if they had a high *P. falciparum* incidence and priority was given to isolated villages close to forests, and those that were participating in malaria prevalence surveys. The art and theatre project took place in 20 villages Battambang during 2016, and in 15 villages in Pailin during 2017. Out of these, 7 villages in Battambang and 3 villages in Pailin were part of the malaria elimination study.

In each village there was a two and a half-day workshop led by the drama team with a public performance on the third evening. During the workshops, participants from different age groups were given singing and drama training as well as education about malaria. A handful of older children were selected to perform alongside professional actors. Others participated in malaria- and vaccination-themed drawing and singing competitions. Villagers contributed real local stories about malaria which were then integrated into the drama performance. The local school was the centre for these workshops and activities, and the final performance was at the village square or temple.

Activities on the third evening, usually lasting from 6 pm to 11 pm, included singing competitions, quizzes with prizes, and a drama performance. Children’s drawings were photographed and projected onto a screen. Videography including drones recorded events and after every performance social media was used to disseminate the activities: facebook.com/dramaagainstmalaria.

## Reflections, challenges and lessons learnt

This section describes the challenges and lessons learnt from our project in 2016 (Lim et al., 2017) and 2017. Our learning from 2016 informed the 2017 project.

### Involvement of local people

In 2016, the project was conducted in 20 villages led by MORU staff and involved 300 community members taking part in competitions or performing alongside professional actors. A total of 8620 people attended the performances. In 2017, the project was held in 15 villages. Local people themselves, rather than MORU staff, directed the planning and implementation of the project. MORU staff provided scientific and administrative support.

Nearly 600 local community members, mostly children, performed and 12,410 people attended – many more than in year 2016. The project in 2017 also got wide coverage by national television and newspapers. We learnt the importance of including elements of a community-directed approach, such as having ideas that come from the community and projects that are led by local leaders.

Compared to 2016, local community members were involved more actively with planning the performances and contributed their own ideas, an approach people reported they had never experienced before.

In the 2017 project, local health authorities suggested adding a secondary theme, infant vaccination. This was to urgently address the low uptake of vaccinations in Cambodia, a challenge faced by local health teams at the time. Having the project incorporate an issue equally important locally at the time was very much appreciated.

### Active participation from children and young people

In 2017, 600 children and young people, twice as many as that in 2016 actively participated in the project, either as a contestant or a performer. In some villagers, where there was a lot of interests and talent, no professional actors were in the final performance; all characters were played by children.

We found that the more children participate, the bigger the audiences for the final show. In addition, we envisage that more active participation – being a performer or contestant versus being a passive audience member means that there will be a better understanding of the science behind vaccination and the efforts to eliminate malaria in their communities.

### Simple key messages and comedy

Our engagement project had three key messages: (1) to use insecticide-treated nets and repellents, (2) to consult a village malaria worker to get early diagnosis and treatment, and (3) to raise awareness about the risk of malaria in local forests. These messages were incorporated into the script and storyline of the drama. The drama used comedic characters from legends known to most people. Villagers were able to recapitulate the drama storyline and key messages and reported that the messages were straightforward and easy to understand, including for people who were illiterate. A member of the provincial health department commented

If we invite them [villagers] to join the workshop and the workshop only focuses on teaching about the health then they won’t understand much. So, to do education by using the arts is very good. This method makes them happy and then they remember it. Then they can apply what they have seen. And also many people attended the event. However, when we did the workshop about health in the health centre of the local government office, then only people who came to get the services could receive the health education. So our workshop was still limited. So the education by using the drama is more comprehensive and many people like to join the event.

### Location and timing of activities

Villagers advised us that performances in the evenings would be popular as school children, farmers and forest workers would have come home. Attendance rates were high in small villages but not as high as we had hoped for in the largest villages, with populations over 1000 people. This was because these large villages were often spread across scattered hamlets and some families had to walk long distances in the dark as there was no electricity. In future projects, we should take into consideration transport challenges. For larger villagers, it may be necessary to have to have the performances at more than one location.

### Uniqueness of the project

Our project offered a unique experience for children and young people. Drama, dance and art workshops by professionals are few and far between in these remote villagers. Children performing on stage alongside professional actors and seeing themselves in newspapers and on television was an even rarer occurrence.

## Conclusions

Participatory art and theatre events that attract large audiences involving local people in the performances, and building health messages inside the scripts and songs, can be an approach for health education in rural areas. We learnt the following lessons from our experience in Cambodia: involving local people including children from the beginning of the project and throughout the process is important; messages should be kept simple; it is necessary to take into consideration practical issues such as location and timing of the activities; and that the project should offer something unique to communities.

## Ethical considerations

The project was done in parallel to a clinical research study approved by the Oxford Tropical Research Ethics Committee (OXTREC; 1015-13), National Ethics Committee for Health Research Cambodia (18th Feb 2016 NECHR 0051), and registered on clinicaltrials.gov (NCT01872702). Verbal informed consent was obtained from all participants who were interviewed regarding the drama.
